# Regional differences in health screening participation between before and during COVID-19 pandemic

**DOI:** 10.1265/ehpm.22-00239

**Published:** 2023-01-26

**Authors:** Yeaeun Kim, Jongho Park, Jae-Hyun Park

**Affiliations:** 1 Department of Health Care Management, Catholic University of Pusan, Busan, South Korea; 2 Division of Health Administration, Gwangju University, Gwangju, South Korea; 3 Department of Social and Preventive Medicine, Sungkyunkwan University School of Medicine, Suwon, South Korea

**Keywords:** Health screening, Health screening participation, COVID-19, Regional vulnerabilities, Geographically weighted regression, GWR

## Abstract

**Background:**

Health screening is a preventive and cost-effective public health strategy for early detection of diseases. However, the COVID-19 pandemic has decreased health screening participation. The aim of this study was to examine regional differences in health screening participation between before and during COVID-19 pandemic and vulnerabilities of health screening participation in the regional context.

**Methods:**

Administrative data from 229 districts consisting of 16 provinces in South Korea and health screening participation rate of each district collected in 2019 and 2020 were included in the study. Data were then analyzed via descriptive statistics and geographically weighted regression (GWR).

**Results:**

This study revealed that health screening participation rates decreased in all districts during COVID-19. Regional vulnerabilities contributing to a further reduction in health screening participation rate included COVID-19 concerns, the population of those aged 65+ years and the disabled, lower education level, lower access to healthcare, and the prevalence of chronic disease. GWR analysis showed that different vulnerable factors had different degrees of influence on differences in health screening participation rate.

**Conclusions:**

These findings could enhance our understanding of decreased health screening participation due to COVID-19 and suggest that regional vulnerabilities should be considered stringent public health strategies after COVID-19.

## Introduction

Health screening is a preventive health strategy for early detection of diseases to identify risks of premature death or severe complications. It is well-known as a cost-effective public health intervention [1, 2]. Recommendations for health screening differ by countries. The World Health Organization commonly emphasizes participation in a timely manner [3]. Since 1980, the Korean government has launched nationwide health screening program and expanded its target diseases and population based on life cycle [4]. It was estimated that approximately 74.1–78.6% of the eligible population had participated in the health screening program for the past 4 years (2016–2019) [5].

The COVID-19 pandemic has decreased preventive healthcare utilization including health screening [6]. In South Korea, only 67.5% of adults participated in the general health screening program during the COVID-19 pandemic in 2020, that was the lowest level in the past five years (2016–2020) [5]. Mularczyk-Tomczewska reported that 30–45% respondents had not participated in basic screening tests during the COVID-19 because COVID-19 reduced the access to preventive healthcare services [7]. In many countries, cancer screening programs were paused or disrupted due to the COVID-19 pandemic. Decreased health screening participation might lead to increased morbidity and mortality rates, resulting in higher burden of disease and medical expense [8–10].

Previous studies have identified individual factors to address health screening participation. Among Korean adults, people in their twenties, those with lower education, and those with lower income levels had lower health screening participation rates [11, 12]. In Poland, sociodemographic factors including older age, higher education level, residence in cities, having at least one chronic disease, and visiting a doctor recently were significantly associated with a higher compliance with preventive health screening [7]. However, recent decrease of health screening participation due to COVID-19 and its affecting factors have not yet been sufficiently addressed. Moreover, although it is clear that health screening participation is an individual’s health behavior, there are significant regional variations. Region-specific factors affecting health screening participation have been reported, including accessibility of health care service, health resources, and regional socioeconomic status [13–15].

The importance of regional parameters in explaining health of populations has been widely demonstrated. Recently, studies have explored the nexus of health and regional factors. Efforts are underway to identify regional cluster and vulnerability of COVID-19 incidence [16–19]. Ulimwengu & Kibonge have suggested that higher vulnerability is associated with higher number of confirmed COVID-19 cases [20]. Vulnerability indices are currently used to assess effects of pre-existing vulnerability factors on COVID-19 infection at a community level, such as Area Deprivation Index (ADI), Social Vulnerability Index (SVI), COVID-19 Community Vulnerability Index (CCVI), and COVID-19 Pandemic Vulnerability Index (PVI) [19, 21–23]. These indices have some component metrics in common, including population density, epidemiological factors, health care system, and high-risk environmental factors.

The aim of this study was to investigate the dynamics of health screening participation in the regional context, which has gone unremarked sufficiently in previous studies. Spatial analytics such as Geographical Weighted Regression (GWR) are critical approaches to statistically examine the geographic relationship between several explanatory variables and outcomes. GWR is a powerful tool that expands the general linear regression and allows regression coefficients to change across space. It is widely used to understand the relationships between regional factors and specific health outcomes, for example, mortality, diabetes prevalence, and pulmonary disease [24–26]. Thus, we examined regional differences in health screening participation between before and during the COVID-19 pandemic and the spatial models were used to determine how well they could explain the regional difference of health screening participation in the South Korea based on several regional vulnerability factors. To the best of our knowledge, this study provides the first attempt to use geographic modeling of health screening participation differences due to COVID-19. It can be insightful for public health policymakers in preparation for targeted interventions.

## Materials and methods

### Data source and preparation

In 2020, there were 229 districts consisting of 16 provinces in South Korea. For this study, we retrieved administrative data from various sources to identify regional factors associated with health screening participation rate before and during the COVID-19 pandemic, such as 2020 Population Census of Ministry of the Interior and Safety, 2019 Infrastructure, and Transport, land ownership of Ministry of Land, 2018 Traffic accessibility Indicator of Korea Transport Institute, 2019 Regional Healthcare Use Statistics of National Health Insurance Corporation, and 2020 Community Health Survey of Korea Centers for Disease Control and Prevention and COVID-19 Statistics of Korean Government. Health screening participation rate of each district was obtained from National Screening Program Statistics in 2019 and 2020 by National Health Insurance Corporation (NHIC). All variables were prepared at the level of 229 districts and joined with corresponding districts in ArcGIS analysis.

### Variables

The dependent variable was rate difference in health screening participation between before and during COVID-19, which was calculated as the difference between community health screening participation rates of 2020 (i.e., during COVID-19) and 2019 (i.e., before COVID-19). Community health screening participation rate was as a proportion of the population participated in the health screening among eligible population of general health screening. As explanatory variables, indicators were presented by five dimensions corresponding to regional vulnerability factors on COVID-19 infection at a community level. Indicators for each dimension were selected as collectable in South Korea (Table 1).

**Table 1:** List of explanatory variables

**Dimension**	**Indicator**	**Data Source (year)**
COVID-19-specific	Total number of populations of cumulative confirmed cases in 2020 (N)	• 16 Local Government webpages (e.g., Seoul Metropolitan Government https://www.seoul.go.kr) (2021)
	Number of populations of cumulative confirmed cases per 100,000 population in 2020 (N)	
	Daily life impact due to the COVID-19 pandemic (average score)	• Korea Centers for Disease Control and Prevention, Community Health Survey (2020)
	Psychological impact due to the COVID-19 pandemic: concerns about infection itself (%)	
	Psychological impact due to the COVID-19 pandemic: concerns about damage caused by infection (%)	
	Psychological impact due to the COVID-19 pandemic: concerns about economic damage caused by infection (%)	
Population	Population density (N/km^2^)	• Minister of Land, Infrastructure, and Transport, land ownership (2019) • Ministry of the Interior and Safety, Resident registration population and generation status (2020)
	Estimated percent of population aged 65+ (%)	
	Estimated percent of population aged 20–39 (%)	
	Estimated percent of population disabled (%)	
Socioeconomic	Average of national health insurance premium (Won)	• National Health Insurance Corporation, regional healthcare use statistics (2019)
	Estimated percent of basic livelihood benefit recipients (%)	• Korea Social Security Information Service (SSiS) (2020)
	Estimated percent of education below high school (%)	• Korea Centers for Disease Control and Prevention, Community Health Survey (2020)
Underlying Health Issues	Prevalence of hypertension among adults aged 30+ (%)	• Korea Centers for Disease Control and Prevention, Community Health Survey (2020)
	Prevalence of diabetes among adults aged 30+ (%)	
	Prevalence of obesity (%)	
	Healthy living practice rate (%)	
Healthcare Infrastructure	Number of health screening facilities per 100,000 population (N)	• Korean government, COVID-19 control homepage (2020)
	Number of hospital beds per 100,000 population (N)	• National Health Insurance Corporation, Health Insurance Review & Assessment Service, Health Insurance Statistics (2019)
	Number of physicians per 100,000 population (N)	
	Number of nurses per 100,000 population (N)	
	Average time to access the nearest general hospital by car (min)	• Korea Transport Institute, traffic accessibility Indicator (2018)
	Average time to access the nearest general hospital by public transportation or on foot (min)	

COVID-19-specific indicators were calculated as average scores or percentages for 229 districts. ‘Daily life impact due to the COVID-19 pandemic’ was the average of scores measured on a 10-point scale ranging from 0 (completely impacted or stopped) to 100 (no impacted or changed) in the daily life of the residents in 229 districts. Each indicator about the psychological impact due to the COVID-19 pandemic including concerns about infection itself, concerns about damage caused by infection, and concerns about the economic damage caused by infection was estimated as the percent of the population who answered ‘strongly agree’ and ‘agree’.

### Data analysis

Descriptive statistics were utilized to identify differences in the health screening participation rate by 229 districts in South Korea. The Global Moran’s index (Moran’s I) was used to measure spatial autocorrelation. The closer Moran’s I to +1 means a positive autocorrelation because values in one area are similar to those in neighboring areas. To investigate the statistical significance of the Moran’s I statistic, Z-test was done at 95% confidence level. Correlations between variables were analyzed, and the Ordinary Least Square (OLS) regression was employed for exploring explanatory variables suitable for the geographically weighted regression (GWR) model. The OLS approach with Variance Inflation Factor (VIF) can detect multicollinearity between redundancy among explanatory variables. Values of VIF exceeding 10 are often regarded as indicating multicollinearity among the explanatory variables. A stepwise selection method, combined forward-backward procedure, was applied to select the variables from the explanatory variables, which are the most optimal for the GWR model. Then, we investigated regional cluster and vulnerability of health screening participant difference by GWR. The corrected Akaike Information Criteria (AICc) and adjusted R-squared were utilized for model comparison of OLS and GWR model. The Parameters of the GWR model were estimated adaptive kernel whose bandwidth was found by minimizing the AICc value. Descriptive, correlation and OLS regression analyses were performed using SAS 9.4. GWR analysis was conducted using ArcGIS pro 2.6.0.

## Results

### Health screening participation rate difference between before and during COVID-19 and its regional distribution in 229 districts

**Fig. 1:**
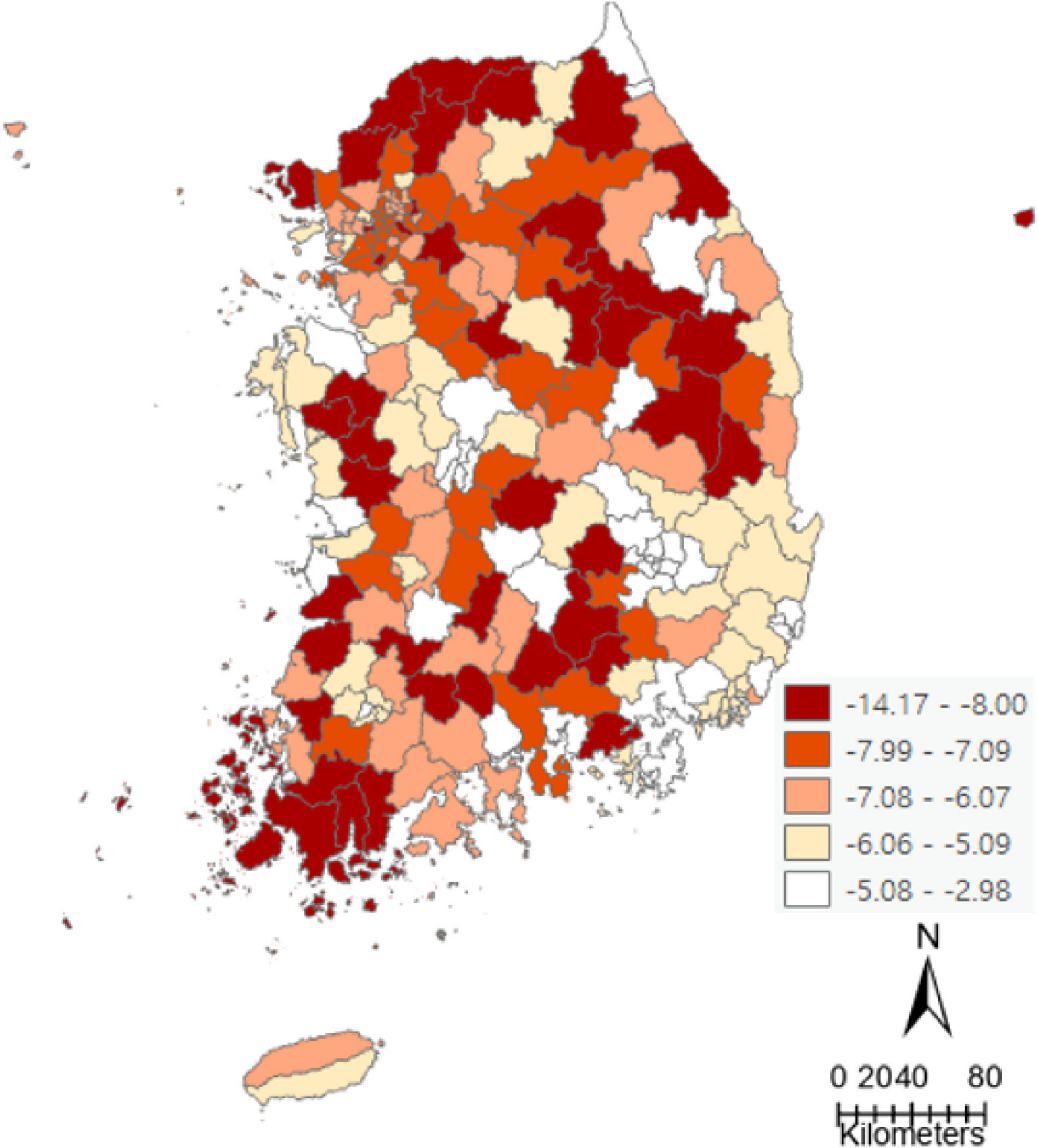
Regional distribution of health screening participation rate difference (2020–2019).

Table 2 shows descriptive statistics of health screening participation rate difference between 2020 and 2019. Compared to that before COVID-19, the health screening participation rate decreased in all districts during COVID-19 (Fig. 1).

**Table 2:** Descriptive statistics of health screening participation rate differences between 2020 and 2019

	**Districts**	**Median**	**Min**	**Max**
Differences in Health screening participation rate (Rate of 2020 − Rate of 2019)	229	−6.64	−14.17	−2.98

### Spatial autocorrelation of health screening participation rate difference (Global Moran’s I)

**Fig. 2:**
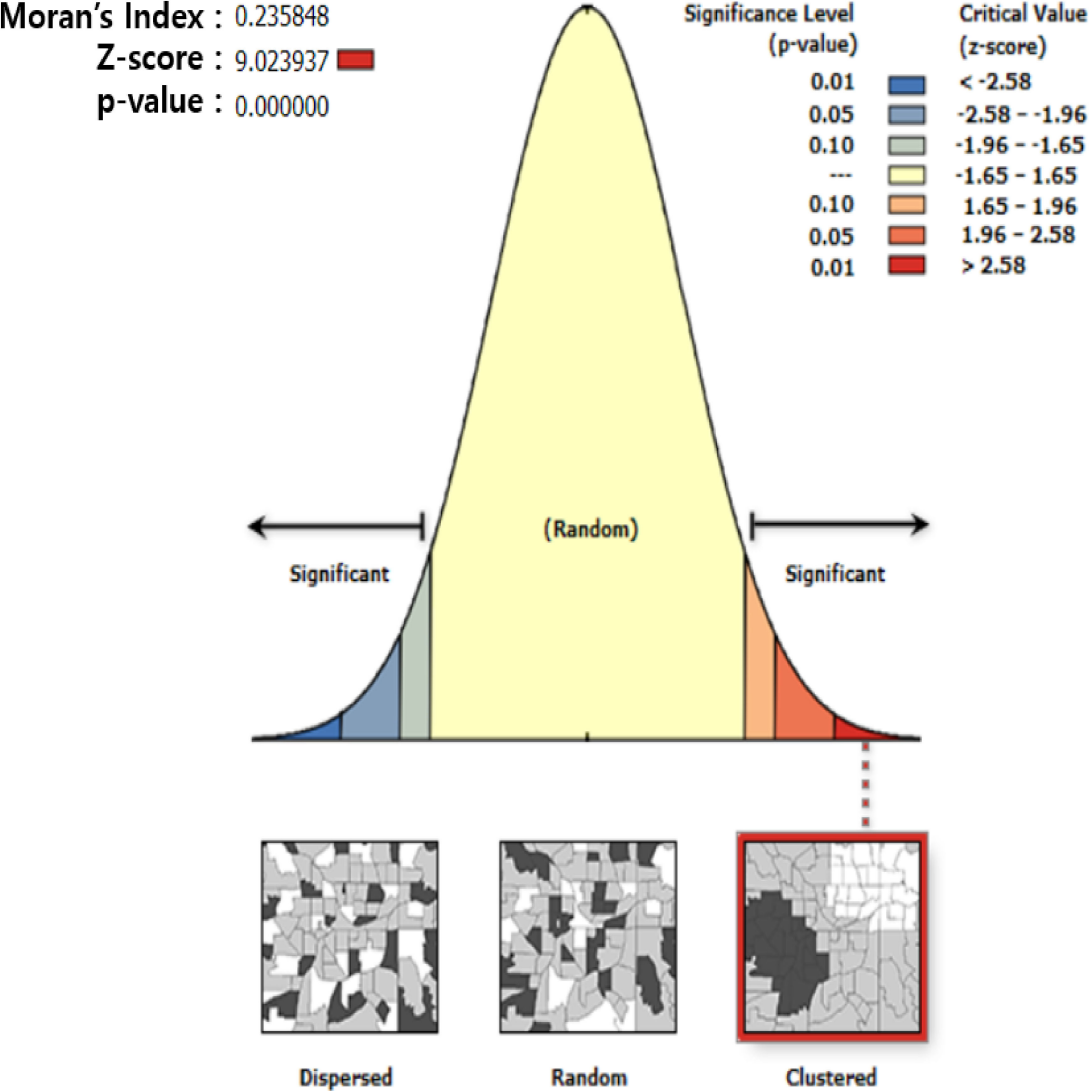
Moran’s diagram for health screening participation rate difference.

The Moran’s index for the health screening participation rate gap between 2020 and 2019 was 0.235848, implying a moderately positive spatial autocorrelation (Fig. 2). This indicated clustering of close values of the health screening participation rate gap (high with high; low with low).

### Correlation analysis

Table 3 summarizes correlation between health screening participation rate gap and regional characteristics. Correlation analysis results showed that the degree of reduction in the health screening participation rate due to COVID-19 was decreased when the population aged 20–39 years, national health insurance premium, health screening facilities, hospital beds, and health care personnel were increased. The degree of reduction, on the other hand, was further increased when COVID-19 concerns, the population aged 65+ and disabled, lower education level and access to healthcare and prevalence of chronic disease were increased. All significant results had p-values less than 0.05 (p < 0.05).

**Table 3:** Correlations between variables

**Variables**		**District (N)**	**Pearson’s correlation coefficient**	**p-value**
Health screening participation rate gap (2020–2019)		229	1	
COVID-19-specific				
	Total number of populations of cumulative confirmed cases in 2020 (N)	229	0.114	0.085
	Number of populations of cumulative confirmed cases per 100,000 population in 2020 (N)	229	0.018	0.790
	Daily life impact due to the COVID-19 pandemic (score)	229	−0.125	0.059
	Psychological impact due to the COVID-19 pandemic: concerns about infection (%)	229	−0.122	0.066
	Psychological impact due to the COVID-19 pandemic: concerns about damage caused by infection (%)	229	−0.199	0.002
	Psychological impact due to the COVID-19 pandemic: concerns about economic damage caused by infection (%)	229	−0.067	0.315
Population				
	Population density (N/km^2^)	229	0.098	0.141
	Estimated percent of population aged 65+ (%)	229	−0.354	<0.001
	Estimated percent of population aged 20–39 (%)	229	0.271	<0.001
	Estimated percent of population disabled (%)	229	−0.322	<0.001
Socioeconomic				
	Average of national health insurance premium (Won)	229	0.233	<0.001
	Estimated percent of basic livelihood benefit recipients (%)	229	0.043	0.516
	Estimated percent of education below high school (%)	229	−0.282	<0.001
Underlying Health Issues				
	Prevalence of hypertension among adults aged 30+ (%)	229	−0.219	0.001
	Prevalence of diabetes among adults aged 30+ (%)	229	−0.139	0.036
	Prevalence of obesity (%)	229	−0.127	0.054
	Healthy living practice rate (%) - non-smoking, moderate drinking, and walking	229	0.103	0.120
Healthcare Infrastructure				
	Number of health screening facilities per 100,000 population (N)	229	0.287	<0.001
	Number of hospital beds per 100,000 population (N)	229	0.229	<0.001
	Number of physicians per 100,000 population (N)	229	0.208	0.002
	Number of nurses per 100,000 population (N)	229	0.241	<0.001
	Average time to access the nearest general hospital by car (min)	229	−0.379	<0.001
	Average time to access the nearest general hospital by public transportation or on foot (min)	229	−0.423	<0.001

### GWR analysis

**Fig. 3:**
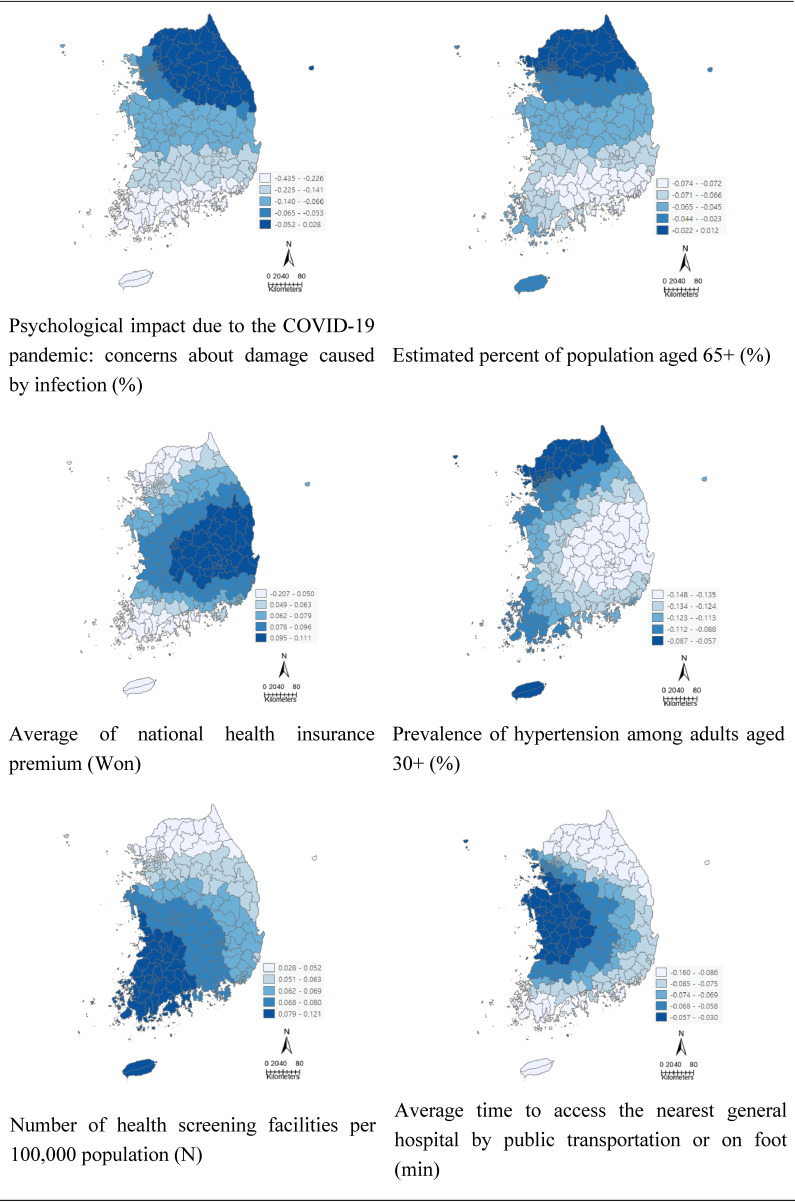
GWR model coefficients for the six selected variables in 229 districts.

Six critical variables were found to be correlated with further reduction in health screening participation rate from correlation analysis and the OLS model, and their coefficients are presented in Table 4. Coefficients of psychological concerns about damage caused by COVID-19 infection had a larger variation than those of other variables. Regions with higher psychological concerns about damage caused by COVID-19 infection, higher percent of population aged 65+, higher prevalence of hypertension, and higher time to access the nearest general hospital by public transportation or on foot had higher reduction in health screening participant rate due to COVID-19. In addition, the reduction in the health screening participation rate decreased even more in regions with lower national health insurance premium and lower number of health screening facilities per 100,000 population. Figure 3 show results of coefficients of GWR for selected variables in describing the geographic distribution of differences in health screening participation rate. It was observed that different factors mentioned as regional vulnerabilities had different degrees of influence on differences in health screening participation rate.

**Table 4:** Summary of coefficient of GWR and OLS model

**Variables**	**GWR Coefficient**			**OLS Coefficient**	**VIF**

	**Median**	**Min.**	**Max.**		
Intercept	−1.821	−5.182	−0.059	−1.997	-
Psychological impact due to the COVID-19 pandemic: concerns about damage caused by infection (%)	−0.094	−0.435	0.028	−0.064	1.164
Estimated percent of population aged 65+ (%)	−0.058	−0.074	0.012	−0.040	2.627
Average of national health insurance premium (Won)	0.073	−0.207	0.111	0.080	2.039
Prevalence of hypertension among adults aged 30+ (%)	−0.120	−0.148	−0.057	−0.122	1.047
Number of health screening facilities per 100,000 population (N)	0.065	0.028	0.121	0.070	1.230
Average time to access the nearest general hospital by public transportation or on foot (min)	−0.072	−0.160	−0.030	−0.061	0.112

Local r-square	0.332	0.169	0.538	-	-

AICc	854.180			884.752	-

Adjusted R-square	0.317			0.214	-

## Discussion

The aim of this study was to explore differences in health screening participation between before and during the COVID-19 pandemic at 229 districts in South Korea. Spatial analytical approach, specifically, geographical weighted regression known to be efficient in identifying the spatial variations and affecting factors, was employed in this study. Twenty-three explanatory vulnerability variables that could potentially affect the health screening participation were categorized under five dimensions: COVID-19-specific, population, socioeconomic, underlying health issues, and healthcare infrastructure.

Health screening participation rate decreased across all regions during the COVID-19 pandemic. It dropped by up to 14.17% compared to the previous year (i.e., before COVID-19). It was consistent with previous studies observing large reductions in the use of preventive health services during the COVID-19 pandemic [27, 28]. For health screening services that require in-person meetings, these declines were related to the social distancing policy that limited human exposure to decrease the spread of the virus. In addition, it might be related to changes in health care system that has been focused on response to COVID-19.

For this study, the GWR model was considered as the best-fitted model for the data since it had the lower AICc value and higher adjusted R-squared value than those in OLS model. The results of GWR analysis demonstrated that the degree of decrease in health screening participation varied from district to district, with regional vulnerabilities showing a greater effect on the reduction of health screening participation in regions. Specifically, increased psychological concerns to COVID-19, the aged structure of the population, lower socioeconomic status, more prevalence of chronic disease, and lower access to healthcare all exhibited influences on the decrease of health screening participation at a regional level. Rather than the COVID-19 infection itself, psychological concerns about damage caused by infection decreased health screening participation. Concerns were associated with perceived vulnerability of COVID-19. There were a wide range of concerns, including hospitalization, mortality, and social impact of COVID-19, which were not limited to infection or financial impact [29, 30]. In addition, since COVID-19 has produced stigma and discrimination that threatens social living in the community, it leads the community to avoid health screening participation [31, 32].

We found that a decrease in the participation rate for health screening was remarkable in districts with a high proportion of the 65-year-old population, hypertension prevalence, and lower economic status. These characteristics identified as regional vulnerability have a negative impact on public health as well as COVID-19 [16, 33]. This result implies that the effect of regional vulnerability reaches a greater impact on more vulnerable regions during the public health crisis. Although the elderly and those with chronic diseases were more likely to participate in the health screening program at the individual level, there could be continued negative effects at the regional level beyond individual factors. Regarding healthcare access, results revealed a significant decrease in districts with fewer health screening facilities and poor access time before and during COVID-19. This was different from previous findings reporting no significant effect of the number of screening centers per 1,000 inhabitants on health screening participation [13]. Due to Korea’s high accessibility to healthcare, it did not significantly affect screening participation under ordinary circumstances without disaster, but it appears to have a greater impact during public health crises with limited access to healthcare [34]. The possible explanation for this is that local residents tend to rely more on local healthcare in disaster situations, so policy alternatives are required to prevent a decrease in the health screening in districts with low healthcare access during these times. Furthermore, no significant reduction was observed in districts with high population density, which were found to be the most susceptible to the outbreak of COVID-19 [35].

Since the spatial model of participation in health screening was not considered sufficiently in recent studies, this study explored an unbalanced distribution of decreased health screening participation and the complex relationship between participation and its vulnerable factors using GWR. However, this study has some limitations. The main limitation of this study was the nature of the ecological and cross-sectional study, therefore the effects of regional factors cannot be interpreted as individual factors, and a causal association between the decrease in health screening participation and the regional vulnerabilities cannot necessarily be ascribed. A further limitation was data availability. Some of the data used as explanatory variables were backdated or not updated, even though they were the most up-to-date data available to the public at the time of the study. Since Moran’s I did not show a strong correlation (0.235848), no significant results were presented for hot spots, which were districts with high differences in health screening participation rates, and cold spots, which were those with low differences. In addition, this study did not reflect contextual issues such as paused or disrupted program operation which could cause limited access to health screening programs during the COVID-19 pandemic. Therefore, inclusion of operation changes of health screening programs over time might deliver slightly better output. Future studies should reflect various temporal factors that might affect health screening participation. Lastly, the limitation of ArcGIS used for GWR analysis should be acknowledged not providing information about interval estimates.

## Conclusion

Given the decreasing participation in health screening after COVID-19, findings of this study can enhance our understanding of decreased health screening participation due to COVID-19, its vulnerable factors, and the extent to which they are affected in each district. Although health screening participation is an individual-level behavior, it is influenced by the environment of the community to which the individual belongs. Therefore, stringent public health strategies are needed to improve regional vulnerabilities in each community after COVID-19. Other public health efforts are also needed from a long-term perspective. Along with that, joint interventions of spatially adjacent regions rather than targeting a single region are recommended to improve participation rate effectively. These approaches could help us implement better health screening program operation and public health decision for allocation of national resources.

## Declarations

### Availability of data and materials

The datasets generated during and/or analyzed during the current study are available from the corresponding author on reasonable request.
